# The effect of life events on NSSI: the chain mediating effect of sleep disturbances and PLEs among Chinese college students

**DOI:** 10.3389/fpsyg.2024.1325436

**Published:** 2024-03-14

**Authors:** Hongcai Wang, Xianhua Liu, Zihao Zeng, Shuangjin Liu, Qin Yang, Qi Qi, Tong Wu, Yiqiu Hu

**Affiliations:** ^1^School of Educational Science, Hunan Normal University, Changsha, China; ^2^School of Electrical Information Engineering, Hunan Institute of Technology, Hengyang, China; ^3^College of Educational Science, Hengyang Normal University, Hengyang, China; ^4^Research Center for Mental Health Education of Hunan Province, Changsha, China; ^5^Cognition and Human Behavior Key Laboratory of Hunan Province, Changsha, China

**Keywords:** life events, NSSI, sleep disturbances, PLEs, college students

## Abstract

This study aimed to explore the relationship between life events and non-suicidal self-injury (NSSI) in college students, as well as the mediating effect of sleep disturbances and psychotic-like experiences (PLEs). After excluding invalid questionnaires, 5,754 were retained, and the valid efficiency was 75.94%. The subjects were aged 16 to 29 years (M = 19.166; SD = 1.392), with 1,969 males (34.22%) and 3,785 females (65.78%). Life events, sleep disturbances, PLEs, and NSSI were assessed using standard scales. Data were analyzed by Pearson Correlation Analysis and bias-correction percentile Bootstrap method. The results show that (1) life events were significant positive predictors of NSSI, sleep disturbances, and PLEs; (2) sleep disturbances, PLEs, and the chain mediation between the two, were mediators between life events and NSSI. Life events are thus shown to be an important external factor influencing NSSI in university students, and this process is mediated through sleep disturbances, PLEs, and the chain between the two. Interventions for NSSI can therefore be made by improving college students’ sleep quality and reducing PLEs.

## 1 Introduction

Non-suicidal self-injury (NSSI) refers to the intentional behavior of individuals who injure themselves without suicidal intent, such as cutting, burning, or scratching themselves (Whitlock et al., 2011; Kiekens et al., 2019). This behavior can occur throughout the course of an individual’s life, however data has shown that the global self-injury rate for college students is 17 to 38% (Whitlock et al., 2011). While the extent and fatality of NSSI may differ among individuals and demographic groups, numerous studies have established its correlation with suicidal tendencies, psychological distress, and various manifestations of mental illness (Yates, 2004; Jacobson and Gould, 2007; Klonsky, 2007). As such, how NSSI develops and its influencing factors demand careful exploration, as understanding the characteristics of NSSI can allow for the development of interventions and preventions.

Life events and NSSI are closely related. According to Nock’s integrated theoretical model of self-injury, self-injurious behavior is an effective way to quickly regulate aversive emotional experiences or social situations, which can be predicted by life events (Nock, 2009). College students are in the process of emerging into adulthood, continuing role exploration which first began in adolescence as they began to take on increasingly adult responsibilities and commitments, and are more likely to experience stressful life events which can lead to psychological problems (Acharya et al., 2018). Compared to those who do not engage in NSSI, self-injurers demonstrate higher physiological arousal in the face of stressful life events, increased emotional distress, and are more likely to adopt self-injury strategies to deal with this resulting emotional distress (Nock, 2010; Riley et al., 2015). Thus, stressful life events are an important factor in individuals’ NSSI. Indeed, stressful life events have been shown to predict NSSI (Lan et al., 2019). Therefore, this study hypothesized that life events predict NSSI.

Sleep disturbances can manifest in a variety of ways, such as difficulty falling asleep, difficulty maintaining sleep, drowsiness, early awakening, and nightmares (Bernert et al., 2015). Moreover, many factors, such as one’s life history or emotional state, can affect one’s sleep (Benjamins et al., 2017). Many studies have shown that stressful life events, such as an individual’s life history, can affect sleep quality, and lower quality sleep is associated with higher levels of self-harming behavior (Gardani et al., 2022). From the clinical perspective, the stress response system plays a vital role in insomnia. Corticotropin-releasing hormone, which regulates sleep and increases under stress, is the leading cause of insomnia (Krueger and Majde, 2003), and animal experiments have shown that using corticotropin-releasing hormone inhibitors reduces the animals’ arousal times (Chang and Opp, 1998). Yoo et al. found that instability of the inhibition of the medial prefrontal cortex on the amygdala and adjacent mesencephalic limbic structures in individuals suffering from insomnia, which resulted in a less-functional emotional response and lower impulse inhibition, further leading to a high correlation between sleep disturbances and self-harm (Yoo et al., 2007). Liu et al. (2016) investigated 2,090 adolescents and found that those with low sleep quality had a higher risk of NSSI. Similarly in adults, individuals with increased insomnia or nightmares demonstrate more self-injurious behaviors (Ennis et al., 2017). As it appears that life events may impact NSSI through sleep disturbances, this study hypothesized that sleep disturbances play a mediating role between life events and NSSI.

Psychotic-like experiences (PLEs) may mediate the relationship between life events and NSSI. PLEs are symptoms similar to those of a clinical psychiatric syndrome (Koyanagi and Stickley, 2015). These PLEs persist due to a variety factors, but can eventually evolve into a mental illness (van Os et al., 2008). Therefore, PLEs are considered to be critical early signs of psychiatric disorders. The cognitive model of integrated social development of mental illness holds that factors such as genetics and childhood adversity influence the development of one’s dopamine system, making it more sensitive, while individuals’ cognitive patterns become paranoid in the face of social adversity. When exposed to stressful life events, these individuals are prone to the dysregulation of dopamine release, which results in the individual experiencing even more stress under the effect of their subsequent irrational cognitive patterns, which leads to a vicious cycle of stress driving dopamine dysregulation, which then generates more stress, further dysregulating dopamine release, and reinforcing paranoid cognitive patterns (Howes and Murray, 2014). Empirical research has also shown that life events are related to increased risk of mental illness (Beards et al., 2013). PLEs are also understood to be an important predictor of NSSI. One meta-analysis found that individuals experiencing PLEs had three times the risk of self-injurious behavior than those who did not experience PLEs (Honings et al., 2015). Moreover, the more frequent the PLEs, the more likely the occurrence of self-injurious behavior, which could be related to negative coping styles (Lee et al., 2021). With this in mind, the current study hypothesized that life events aggravate one’s experience of PLEs and promote NSSI, which is to say that PLEs play an intermediary role between life events and NSSI.

**Figure 1:**
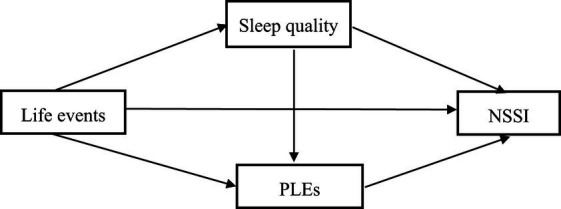
Chain mediating model hypothesis between sleep quality and PLEs in the relationship between life events and NSSI.

Meanwhile, sleep disturbances may mediate the relationship between life events and PLEs. The diathesis–stress conceptualization of schizophrenia suggests that sleep plays a critical role at multiple time points in neurodevelopment, and that sleep disturbances increase one’s stress response to stressful events, and can exacerbate the vicious cycle of psychotic episodes (Lunsford-Avery and Mittal, 2013). Marcusson-Clavertz et al. (2022) conducted coordinated analyzes of the data from several studies which employed ecological momentary assessments to capture naturally-occurring, self-reported stress and sleep, and found that life events as external factors did have an impact on participants’ reported quality of sleep. Furthermore, a series of sleep disturbances have been observed across the entire spectrum of mental illnesses, and the relationship between sleep disturbances and PLEs is shown to have cross-cultural consistency (Lunsford-Avery and Mittal, 2013; Koyanagi and Stickley, 2015). Studies have also found that disruptions to circadian rhythms caused by sleep disturbances are associated with the severity of psychotic symptoms, and may be a pre-defining feature of the emergence of psychosis (Lunsford-Avery et al., 2017). It is thus reasonable to assume that life events affect sleep, and sleep disturbances affect PLEs. Therefore, this study further hypothesized that life events impact self-injurious behavior through a chain-mediating effect of sleep disturbances and PLEs. The resulting chain mediating model hypothesis model is shown in Figure 1.

## 2 Methods

### 2.1 Subject and procedure

A convenience sampling method was used to invite students from five universities located in Hunan Province and Guangdong to complete a questionnaire survey via the online survey website Wenjuanxing.^1^ A total of 7,577 questionnaires were collected. After excluding invalid questionnaires (i.e., item responses were quicker than the reasonable rate of 2 s per item), 5,754 questionnaires were deemed valid (75.940% valid response rate; DeSimone et al., 2014). All subjects were from 16 to 29 years of age (M = 19.166; SD = 1.392), with 1,969 males (34.220%) and 3,785 females (65.780%); 2,430 first-year students (42.231%), 1,388 s-year students (24.122%), 1,225 third-year students (21.290%), 638 fourth-year students (11.088%), and 73 fifth-year students (1.269%).

### 2.2 Measures

#### 2.2.1 Adolescent self-rating life events checklist (ASLEC)

ASLEC was developed by Liu et al. (1997), which consisted of 26 negative stress events including family (e.g., “ignored by parents”), school (e.g., “failure in final exam”), interpersonal (e.g., “break up with a close friend”), and individual (e.g., “serious illness”). Respondents are asked whether they and their family have experienced the events listed in the scale over the course of the previous 3 months. If they have not, the score is 0. If they have experienced it, the respondent rates the item according to event’s degree of impact in their life (1 = no effect, 2 = mild, 3 = moderate, 4 = severe, and 5 = extremely severe). Higher average scores indicate higher levels of stressful life events. The Cronbach’s α for the ASLEC in the current study was 0.941.

#### 2.2.2 Insomnia severity index scale (ISI)

The ISI, as translated into Chinese by Yu (2010), was used to evaluate participants’ severity of insomnia, as this would also provide information regarding the impact of insomnia on their health and daytime functioning. The scale includes seven items, each of which is rated using a Likert scale ranging from 0 (no problem) to 4 (very severe problem). The higher the total score, the more serious the respondent’s insomnia symptoms. In this study, the Cronbach’s α for the ISI was 0.934.

#### 2.2.3 PLEs

The positive subscale of the Community Assessment of Psychological Experiences (CAPE; Mark and Toulopoulou, 2017) was used to measure respondents’ experiences of PLEs in this study. The subscale comprises eight items evaluating two aspects: delusional experiences (delusion of reference, fear that someone wants to kill them, erased thoughts, inserted thoughts, thoughts being broadcast, feeling of being controlled) and hallucinations (e.g., visual or auditory hallucinations). The frequency of PLEs was also measured using one item which asked how often they experienced these thoughts, which was rated on a scale ranging from 1 (never) to 4 (very often). The eight items taken from the CAPE scale have been shown to have high validity in Chinese college students (Wang et al., 2022). In this study, the Cronbach’s α for these items was 0.926.

#### 2.2.4 The deliberate self-harm inventory (DSHI)

The DSHI, as constructed and validated by Gratz (2001), has been shown to have good reliability and validity in measuring adolescents’ self-harm behavior in China (Lan et al., 2019). The questionnaire is a one-dimensional scale with nine items (e.g., “Pierce my skin with sharp objects”). Each item is scored on a six-point scale ranging from 0 (none) to 5 (five or more times). The higher the score, the more serious the adolescent’s self-injury behavior, with a total possible score for the full scale between 0 and 45. In this study, the Cronbach’s α for the DSHI was 0.894.

### 2.3 Data analysis

Data analysis was performed using SPSS 25.0 and PROCESS. To check for potential common method bias, Harman’s single-factor analysis was first conducted. The reliability of each of the scales used in the current study was evaluated using Cronbach’s α coefficient. Pearson correlation coefficients were then calculated to explore the associations between the variables. Subsequently, the PROCESS (Model 6) was employed to investigate the chain mediation relationships among life events, sleep quality, PLEs, and NSSI.

## 3 Results of analysis

### 3.1 Multicollinearity and common method variance

Exploratory factor analysis was used to test for possible common method bias by integrating all questionnaire items. The results showed seven factors with eigenvalues greater than 1. The first factor accounted for 27.720%, which is less than 40% (Tang and Wen, 2020), indicating no serious common method deviation in the data of this study. As gender and region could influence the results, we then used these as control variables for the correlational analysis. The results showed that life events, sleep quality, PLEs, and NSSI were all significantly correlated with one another (see Table 1). The results of the subsequent logistic regression analysis indicated that gender, life events, sleep quality, and PLEs could all positively predict NSSI (see Table 2).

**Table 1:** Descriptive statistics and correlation analysis results.

Variables	M	SD	1	2	3	4
1. Life events	44.296	17.691	1			
2. SQ	13.141	5.675	0.350^***^	1		
3. PLEs	10.695	3.881	0.336^***^	0.485^***^	1	
4. NSSI	9.562	2.795	0.205^***^	0.229^***^	0.257^***^	1

*N* = 5,754; SQ, sleep quality; PLEs, psychotic-like experiences; NSSI, non-suicidal self-injury. ^*^p < 0.05, ^**^p < 0.01, ^***^p < 0.001.

**Table 2:** Results of regression analysis.

Predictors	Adolescent NSSI		
	*β*	*t*	OR (95% CI)
Region	−0.02	−1.44	−0.10, 0.02
Gender	0.03	2.68^**^	0.02, 0.13
Year of study	−0.01	−0.45	−0.03, 0.02
Life events	0.11	8.01^***^	0.00, 0.01
Sleep quality	0.11	7.44^***^	0.01, 0.02
PLEs	0.17	11.38^***^	0.04, 0.05
*R* ^2^		0.09	
*F*		96.63^***^	

NSSI was used as the dependent variable; *β*, standardized coefficient; NSSI, non-suicidal self-injury; OR, odd ratio; CI, confidence interval; PLEs, psychotic-like experiences.^*^p < 0.05, ^**^p < 0.01, ^***^p < 0.001.

### 3.2 The mediating effects of sleep quality and PLEs

A significant correlation was found between life events, sleep quality, PLEs, and NSSI, and the results supported conducting a further mediating effects analysis (Wen and Fan, 2015). Process 3.3 was used to analyze the mediating role of sleep quality and PLEs between life events and NSSI, controlling for gender and year of study. The results of the regression analysis (see Table 3) showed that life events had a significant positive predictive effect on NSSI (*β* = 0.206, *p* < 0.01). When life events, sleep quality, and PLEs were included in the regression equation, the predictive effect of life events on NSSI changed (*β* = 0.111, *p* < 0.001). Life events positively predicted sleep quality (*β* = 0.351, *p* < 0.001) and PLEs (*β* = 0.189, *p* < 0.001); sleep quality positively predicted PLEs (*β* = 0.417, *p* < 0.001) and NSSI (*β* = 0.111, *p* < 0.001); and PLEs positively predicted NSSI (*β* = 0.167, *p* < 0.001).

**Table 3:** Results of the regression analysis of the relationship between model variables.

Predictors	Model 1 (SQ)		Model 2 (PLEs)		Model 3 (NSSI)	
	*β*	*t*	*β*	*t*	*β*	*t*
Region	−0.069	−2.280	0.036	1.289	−0.044	−1.435
Gender	−0.139	−5.217^***^	−0.142	−5.787^***^	0.074	2.684^**^
Year of study	0.085	7.274^***^	0.005	0.451	−0.005	−0.447
Life events	0.349	28.415^***^	0.190	15.759^***^	0.110	8.009^***^
SQ			0.417	34.522^***^	0.110	7.439^***^
PLEs					0.168	11.376^***^
*R* ^2^	0.132		0.272		0.092	
*F*	220.881^***^		428.870^***^		96.626^***^	

The model variables are all standardized. *β*, standardized coefficient; SQ, sleep quality; PLEs, psychotic-like experiences; NSSI, non-suicidal self-injury.

**Figure 2:**
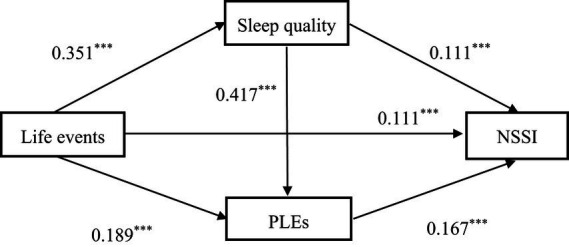
Illustrated pathway showing the influence of life events on NSSI.

The results of the mediated effects analysis showed that sleep quality and PLEs mediate the relationship between life events and NSSI, with a mediated effect value of 0.951. More specifically, the mediating effect consists of indirect effects produced by three pathways: Indirect Effect 1 (0.039), through life events → sleep quality → NSSI; Indirect Effect 2 (0.032), through life events → PLEs → NSSI; and Indirect Effect 3 (0.025), through life events → sleep quality → PLEs → NSSI. Table 4 shows that these three indirect effects account for 18.941, 15.541, and 12.142% of the total effect, respectively, and none of the bootstrapped 95% confidence intervals for these indirect effects contained a value of 0, indicating that all three indirect results reached a significant level. The detailed pathway model is shown in Figure 2.

**Table 4:** The moderating effects of sleep quality and PLEs.

	Effect	Boot SE	Boot LLCI	Boot ULCI	Ratio of indirect to total effect
Total indirect effect	0.095	0.013	0.072	0.123	46.187%
Indirect Effect 1	0.039	0.008	0.025	0.055	18.941%
Indirect Effect 2	0.032	0.006	0.022	0.044	15.347%
Indirect Effect 3	0.024	0.004	0.017	0.034	11.899%
Comparison 1	0.007	0.009	−0.009	0.025	
Comparison 2	0.015	0.008	0.000	0.031	
Comparison 3	0.007	0.003	0.001	0.014	

Boot SE, bootstrap standard error; Boot LLCI, bootstrap lower limit confidence interval; Boot ULCI, bootstrap upper limit confidence interval. Indirect Effect 1, life events→ sleep quality → NSSI; Indirect Effect 2, life events → PLEs → NSSI; Indirect Effect 3, life events → sleep quality → PLEs → NSSI. Standard errors were estimated for Boot SE, Boot LLCI, and Boot ULCL, with lower and upper 95% confidence intervals (calculated through the bias-corrected percentile bootstrap method) used to test for indirect effects. All values are retained to three decimal places.

## 4 Discussion

This study explored the effects of life events, sleep quality, and PLEs on NSSI in college students by constructing a chain mediation model to examine the mediating role of sleep quality and PLEs in the relationship between life events and NSSI. The results indicate a significant positive correlation between life events and NSSI. Further regression analysis results also indicate a significant positive predictive effect of life events on NSSI, thus confirming our first research hypothesis. When sleep quality and PLEs were entered into the equation, the direct predictive effect of life events on NSSI was insignificant. The findings of the mediation test revealed that sleep quality and PLEs fully mediated the effect of life events on NSSI, and that this mediation involves three pathways: a single mediation of sleep quality, a single mediation of PLEs, and a chain mediation of sleep quality to PLEs. To test our additional hypotheses and to better understand the mechanisms of NSSI, the mediating roles of the variables were also tested.

### 4.1 The relationship between life events and NSSI

The results of this study showed that life events predicted NSSI, which is consistent with the findings of previous studies (Lan et al., 2019; Hamza et al., 2021). According to Nock’s integrated theoretical model of the development and maintenance of NSSI, individuals have both personal and interpersonal vulnerabilities that limit their coping abilities in the face of stressful life events, and some people are more likely to adopt inappropriate coping techniques, such as self-injury, to regulate the negative emotions and cognitive experiences that result from these events (Nock, 2010). Life events such as academic stress, failed exams, unsuccessful relationships, or parental conflict have all been shown to influence college students’ NSSI (Macrynikola et al., 2018). Meanwhile, those experiencing NSSI have been shown to have a higher level of emotional reactivity in response to life events as compared to those without NSSI (Lucas et al., 2019; Hamza et al., 2021). As they are in a transition stage between school and grown-up society, yet still have limited problem-solving abilities, college students face numerous life events, and will inevitably experience negative emotions as they struggle or fail to solve certain problems. Many of these college students may engage in NSSI when these negative emotions have built up to a certain level, and seem impossible to eliminate in any other way. However, it may be possible to reduce the likelihood of college students engaging in NSSI by providing them with training to improve their problem-solving skills and to teach them better emotional regulation strategies.

### 4.2 The individual mediation effects of sleep quality and PLEs

The results of this study showed that life events impact the NSSI of college students through the separate mediating effects of sleep quality and PLEs. This suggests that sleep quality and PLEs are important factors in triggering maladaptive psychological adjustments to life events. Sleep is already understood as being essential for the promotion of one’s physical and mental health, and sleep disturbance is therefore a significant threat to numerous physical, psychological, and social functions, impacting one’s cognition, mood, quality of life, learning abilities, and social interactions (Owens et al., 2014). Life events can be a cause of psychological stress, which leads to the activation of the hypothalamic–pituitary-adrenocortical (HPA) axis and increasing one’s arousal, resulting in sleep disturbances (Richardson, 2007). Meanwhile, ruminating thoughts about life events, either conscious or subconscious, will result in individuals focusing even more on the negative emotions and threatening cues triggered by these stressful life events, thereby triggering autonomous arousal and emotional distress in these individuals, leading them to experience sleep disturbances (Gu et al., 2018). Sleep disturbances are strongly associated with NSSI, which can be explained by the fact that sleep plays a positive role in mood stabilization and regulation, and facilitates one’s adaptive emotional responses throughout the following day. Sleep disorders affect one’s emotional stability and impair their ability to use emotional regulation strategies effectively, increasing the likelihood that one might adopt NSSI as a means of emotional regulation.

PLEs were also shown to mediate the impact of life events on NSSI. The results of this study suggest that the more life events one experiences, the more significant their experience of psychoticism, and the more likely they may be to engage in NSSI. The stress susceptibility model for the development of mental illness asserts that every individual had susceptibility factors for mental illness, and everyone copes with life events in a dynamically balanced manner when the stress generated by life events remains under a certain threshold; however, positive symptoms of mental illness can occur when the psychological stress generated exceeds one’s tolerance threshold (Zubin and Spring, 1977). While individuals with low susceptibility factors require a higher intensity of stressful life events to be at risk of developing a mental illness, those with high susceptibility factors may experience mental illnesses even when faced with low-frequency, low-level life event stress. As one experiences more frequent and severe life events, their risk of mental illness becomes greater. Individuals with PLEs already have cognitive biases that predispose them to cognitive weakness, while those with inadequate problem-solving skills will experience greater negative emotions, making them more likely to attempt to relieve their emotions through NSSI.

### 4.3 The chain mediating effect of sleep quality and PLEs

The results of this study found that the sleep quality and PLEs together mediate the relationship between life events and NSSI in college students. In other words, PLEs were shown to mediate the effect of sleep quality on NSSI, and the results are consistent with those of previous studies (Cohrs, 2008). A possible explanation for this could be that disturbed sleep does not alleviate negative emotions and allow one to recover one’s energy (Palmer and Alfano, 2017). This means that individuals will continue to be more sensitive to stressful events (Lunsford-Avery and Mittal, 2013), which will thereby affect their cognitive flexibility (Gobin et al., 2015), making them more likely to experience PLEs and, eventually, NSSI (Howes and Murray, 2014). Furthermore, individuals who are suffering from a higher frequency and severity of life events will become stressed, which then affects their quality of sleep (Benjamins et al., 2017). Conversely, individuals experiencing fewer life events are less likely to suffer from sleep disturbances, which in turn allows their sleep to somewhat alleviate the negative emotions associated with their current life events. This means that individuals in a healthy, strong emotional state are less likely to experience PLEs, and are thus less likely to engage in NSSI.

Altogether, the finding of this study reveal the potential psychological mechanisms at play in how life events affect NSSI in college students. Specifically, life events impact college students’ NSSI through the chain mediating role of sleep quality and PLEs. This finding confirms the mechanisms of the influence of life events on individuals’ psychology, enriching the existing research findings related to NSSI. These findings suggest that the development of interventions on NSSI in college students should focus on sleep quality and PLEs. Furthermore, college administrators or professors should be proactive in NSSI interventions by providing students with a variety of techniques to reduce their sleep problems and PLEs, with the aim of avoiding much more serious stages and consequences later on.

## 5 Contributions and limitations

This study explored the relationship between life events and NSSI, as well as the mechanisms involved. Not only do life events directly cause NSSI in college students, but they were also shown to impact NSSI through the separate mediations of sleep disturbances and PLEs, as well as through the chain mediation of both. These findings provide insight into the mechanisms underlying the influence of life events on NSSI, and serve as a guide for NSSI prevention and interventions. First, life events are important triggers of NSSI in college students. Therefore, we should pay attention to individuals experiencing a significant life event throughout their day-to-day routines. Second, several risk factors (e.g., sleep disturbances, PLEs), which may be pre-existing or secondary to the individual, can exacerbate the impacts of a major life event on the individual. If someone shows signs of suffering from the stressful impacts of life events, everyday risk factors should be minimized or targeted through early interventions, and these interventions should be undertaken as soon as possible to prevent more serious consequences later on.

Despite its contributions to the existing literature, this study does have certain limitations, which should be noted. First, this study used a questionnaire method, which is useful for the analysis of cross-sectional data, however this design makes it difficult to determine causal relationships between variables. A longitudinal study design should be adopted in future research to further validate the findings of this study. Second, our examination of the relationship between life events and NSSI did not include all possible influencing factors, such as the individual’s mood (e.g., suffering from anxiety or depression), impulsivity, and others, which limits the stability of the relationship depiction. Future research should include wider possible influencing factors to enhance predictability. Finally, additional protective factors such as social support, should be considered in future research, to explore the buffering effects of these protective factors against the shock and stress of major life events.

## 6 Conclusion

(1) Life events, sleep quality, PLEs, and NSSI are all significantly correlated, and life events significantly predicts NSSI.
(2) Sleep quality and PLEs significantly mediate the relationship between life events and NSSI. This mediation consists of three pathways: a separate mediation of sleep quality, a separate mediation of PLEs, and a chain mediation of sleep quality and PLEs.

## Data availability statement

The raw data supporting the conclusions of this article will be made available by the authors, without undue reservation.

## Ethics statement

The studies involving humans were approved by the Ethics Committee of Hunan Normal University, China. The studies were conducted in accordance with the local legislation and institutional requirements. The participants and the participants' legal guardians/next of kin provided their written informed consent to participate in this study.

## Author contributions

HW: Conceptualization, Data curation, Writing – original draft, Writing – review & editing. XL: Conceptualization, Conceptualization, Formal analysis. ZZ: Data curation, Methodology, Writing – review & editing. SL: Formal analysis, Funding acquisition, Writing – review & editing. QY: Data curation, Methodology, Writing – review & editing. QQ: Data curation, Writing – review & editing. TW: Data curation, Writing – review & editing. YH: Conceptualization, Formal analysis, Supervision, Writing – original draft, Writing – review & editing.

## Footnotes

^1^www.sojump.com
